# Retrospective Study of Minimal Three-Year Follow-Up of Transforaminal Endoscopic Discectomy for Lumbar Disc Herniation: 5000 Multicenter Cases

**DOI:** 10.7759/cureus.50993

**Published:** 2023-12-23

**Authors:** Chuanchao Du, Kunfeng Song, Bao Hai, Xiangyu Wang

**Affiliations:** 1 Orthopedic Surgery, Emergency General Hospital/National Emergency Medicine Research Center, Beijing, CHN; 2 Center of Minimal Invasive Spine Surgery, Henan People's Third Hospital, Zhengzhou, CHN; 3 Orthopedic Surgery, Peking University Third Hospital, Beijing, CHN; 4 Pain Department, People's Liberation Army General Hospital, Beijing, CHN

**Keywords:** quality of life, inside-out, outside-in, retrospective study, percutaneous transforaminal endoscopiclumbar discectomy (pteld)

## Abstract

Objective

The objective of this study was to investigate the long-term effects of percutaneous transforaminal endoscopic lumbar discectomy (PTELD) and clarify the differences between outside-in and inside-out techniques.

Methodology

This was a multicenter retrospective study with a chart review of questionnaires about patients' quality of life. Patients were recruited from three hospitals in China. Based on certain inclusion and exclusion criteria, we enrolled in the study 5000 patients aged ≥18 years diagnosed with lumbar disc herniation who received PTELD from September 2015 to September 2019. The outside-in technique (n=2039) was compared with the inside-out technique for PTELD (n=1890) on the Visual Analogue Scale (VAS), Oswestry Disability Index (ODI), and the Short Form 36 (SF-36) of the Health Survey Questionnaire (physical component) both pre-operatively and post-operatively.

Results

VAS, ODI, and SF-36 significantly improved just after surgery for both techniques compared with pre-operative status. Nevertheless, significant differences existed between the two techniques concerning VAS for leg pain, VAS for back pain, ODI, and SF-36 at 0.5 months post-operatively. The above indices steadily improved within six months after both techniques, after which they did not significantly improve. In detail, outside-in patients suffered more back pain and worse ODI and SF-36 (physical) but had more relief from leg pain 0.5 months after surgery in terms of VAS. As for recovery rate from symptoms, there were only significant differences in recovery rate for leg pain and back pain at the first 1.5 months post-operatively. As for satisfaction rates, the outside-in technique had better results than the inside-out technique at both 0.5 months and 12 months.

Conclusion

Both techniques could relieve the symptoms of lumbar disc herniation. However, patients in the outside-in group suffered more back pain and a bigger risk of nerve injury than those in the inside-out group.

## Introduction

Lumbar disc herniation (LDH) is one of the most common diseases, affecting approximately one in five adults, who mainly complain of low back pain radiating to the buttocks, back thighs, or calves, even to the feet [1]. Although the severity of symptoms may vary, the Quality of Life Scale (QOLS) scores of patients suffering from lumber disc herniation are obviously reduced. However, fewer than two out of ten patients are required to have discectomy surgery through traditional open discectomy or minimally invasive discectomy. In addition to the pioneering research and development of spine endoscopic instruments, the extensive usage of spine endoscopic technique should be credited to Dr. Kambin, who first put forward a new concept of spinal anatomy - Kambin's triangle [2]. Among the flourishing minimally invasive techniques, percutaneous transforaminal endoscopic lumbar discectomy (PTELD) is the most popular [3]. After much research, Kambin published an article on lateral percutaneous nucleus pulpous removal in 1982. In 1988, when he was 57 years old, he proposed the concept of Kambin's triangle, suggesting that this region was a safe anatomical corridor to access nerve roots and dura sacs, as it is devoid of important vascular and neural structures [4]. The proposal of this concept laid a theoretical foundation for the extensive development of lateral spinal endoscopy. Since then, Yeung from the United States and Hoogland from Germany proposed Yeung endoscopic spine system and transforminal endoscopic spine system technologies, respectively, which served as the prelude to the global popularity of spinal endoscopy [5-7]. However, people remain skeptical about the new technology represented by PTELD due to concerns about a lack of high-level evidence, follow-up, and replicability. Furthermore, although there are a variety of procedures and detailed techniques in PTELD, few studies comparing them have been published [8]. More importantly, there is currently significant variation in the literature regarding the exact indications and procedures, such as entering the disc space first (inside-out) for pure LDH or outside-in for LDH with foraminal stenosis [8-10]. Herein, we present a large-scale multicenter retrospective study of different PTELD procedures and techniques to show their effects and differences.

## Materials and methods

Equity, diversity, and inclusion statement

**Figure 1:**
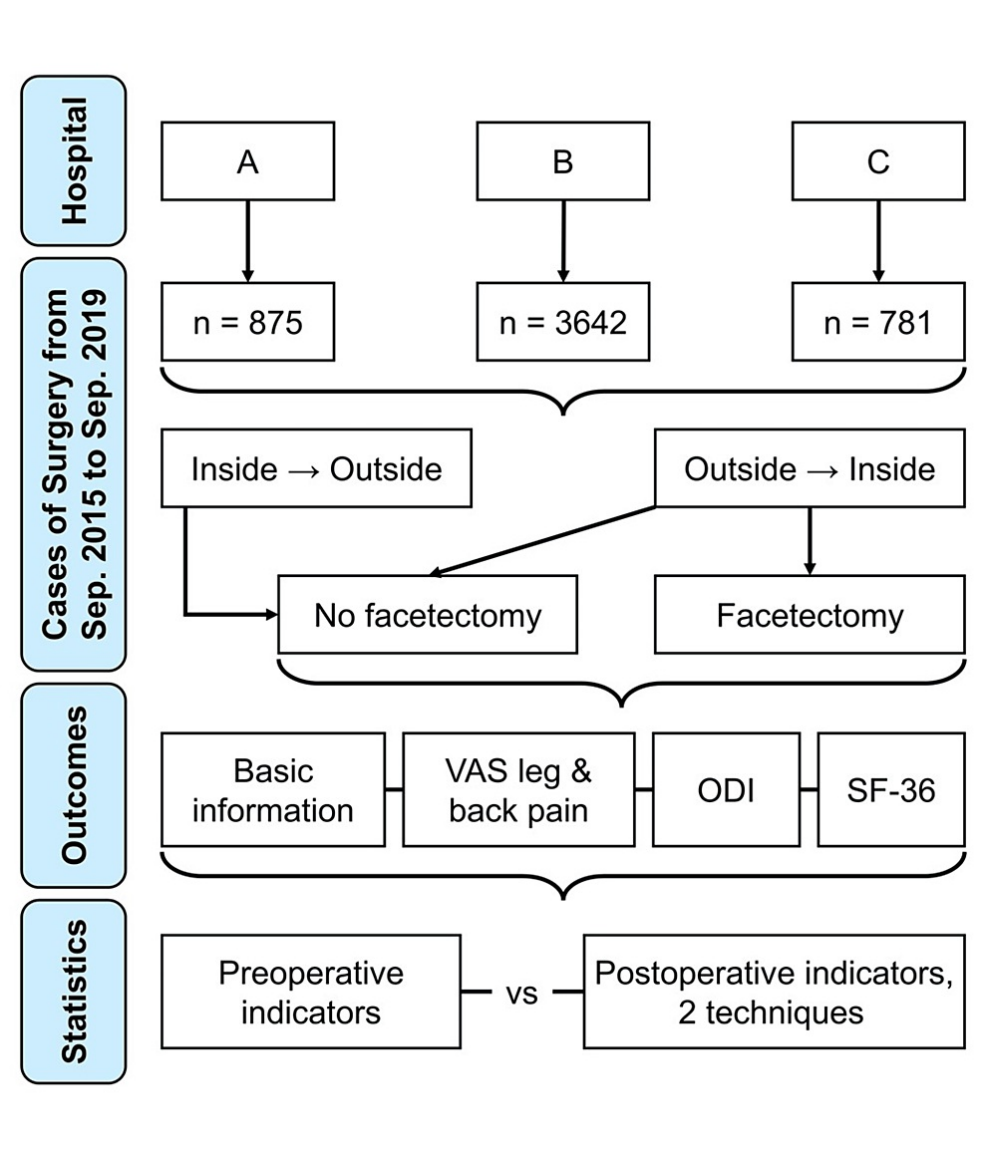
Flow diagram of the retrospective study VAS - Visual Analogue Scale; ODI - Oswestry Disability Index; SF-36 - physical component of Short Form 36 Health Survey Questionnaire

Our research includes women and men (2:3) with lumbar disc herniation from three hospitals in China in a specified period. We did not purposefully recruit people from marginalized communities, as we were concerned that different cultures and health economics may influence the outcomes. We acknowledge that we did not examine the effects of race/ethnicity or socioeconomic status, which may influence the outcomes. A flow diagram of this retrospective study is presented in Figure 1.

Study design

The inclusion criteria of this study were as follows. We included patients diagnosed with LDH and received PTELD, ≥18 years old, and had complete medical files, including pre-operative and post-operative VAS [11], ODI [12], and the Short Form 36 (SF-36) of the Health Survey Questionnaire (physical component) (Scoliosis Research Society, SRS)[13]. We excluded patients diagnosed with concomitant lumbar canal stenosis, spinal tumor, infection, sequela of stroke, cervical and thoracic neuropathy conditions, had a history of spine open surgery, did not have six months of conservative treatments, and were unable to complete questionnaires. Finally, 5000 patients from three hospitals were enrolled from September 2015 to September 2019. There were 875 cases from hospital A, 3642 cases from hospital B, and 781 cases from hospital C. Basic information of the patients (age, gender, body mass index), chief complaints, pre-operative and post-operative ODI, VAS for leg and back, and SF-36 were documented. PTELDs were completed by experienced surgeons who completed >600 cases independently before the beginning of the study. The procedures were usually done with one of two techniques, outside-in and inside-out, as defined by the sequence of removing the disc [8-10]. However, the enrolled surgeons were often better at one of the techniques; therefore, all the enrolled patients were treated by the most experienced surgeons using their preferred technique, as appropriate. Generally, the outside-in technique necessitates the removal of part of the superior articular process (SAP) or facetectomy (undercutting the facets), whereas the inside-out technique does not. In this retrospective study, we compared VAS, ODI, SF-36, and satisfaction rates between the two techniques, as well as the change of function status from pre-operative status to follow-up.

Full-endoscopic lumbar discectomy through transforaminal approach

**Figure 2:**
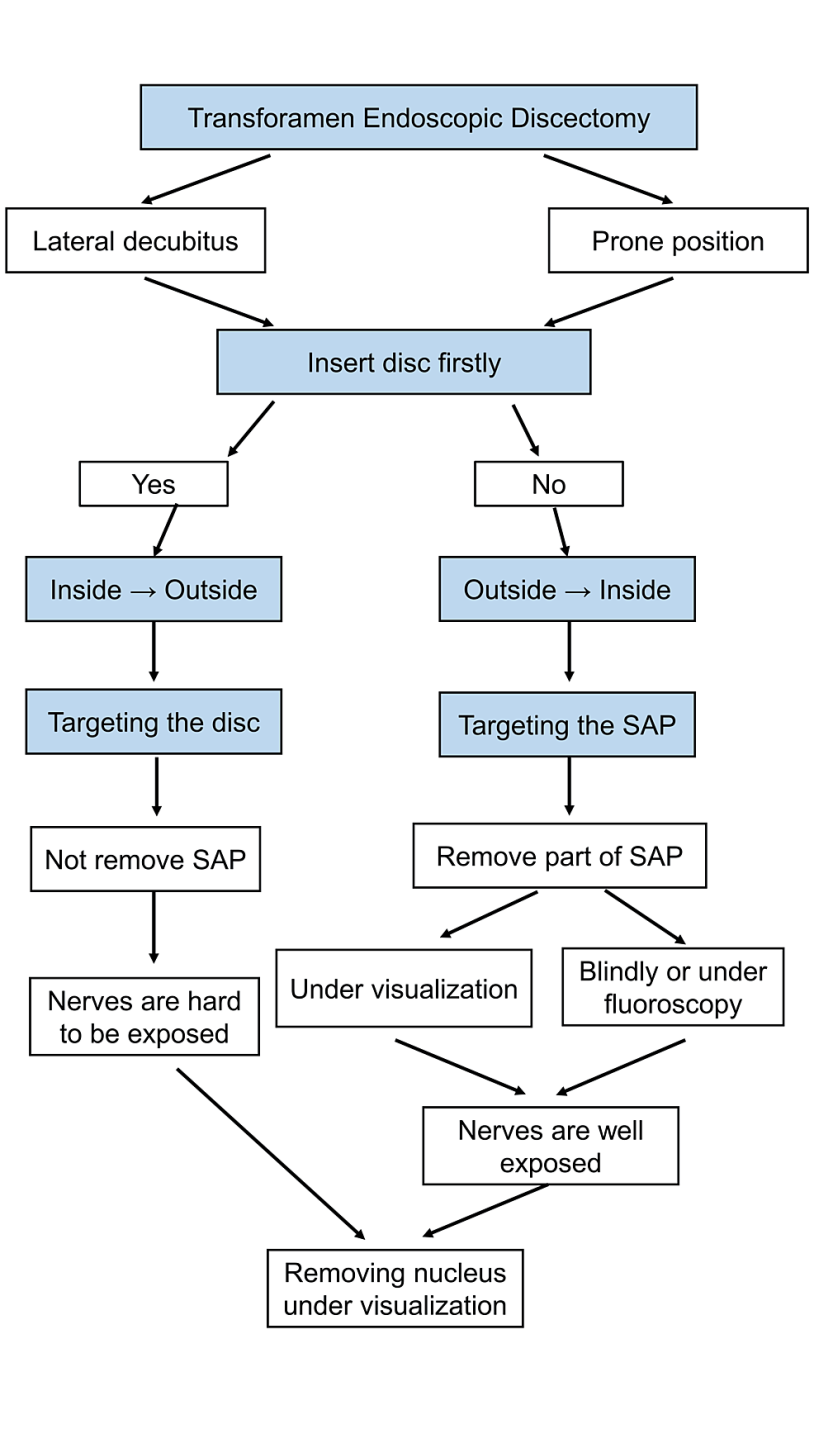
Comparison of inside-out and outside-in techniques SAP - superior articular process

For the PTELD procedures, 1872 patients were placed in the prone position, and 3128 patients were placed in the lateral decubitus position, as decided by the surgeon. C-arm fluoroscopy was used before surgery to draw a projection line of the targeted intervertebral space (targeting reference line), the ventral line of the SAP (sagittal targeting reference line), and the longitudinal line of the posterior border of the vertebral body (sagittal safe reference line). However, 2039 patients received the outside-in technique, and 1890 patients received the inside-out technique, with the difference being whether an intervertebral disc was first inserted: if the disc was first inserted, then the nucleus pulposus was removed from inside to outside, hence named the inside-out technique; conversely, in the outside-in technique, a disc was not first inserted. In the outside-in technique, partial SAP resection (undercutting facetectomy) may be needed to enlarge the foramen space (foraminoplasty), exposing the traversing nerve root and disc annulus [14]. In contrast, in the inside-out technique, disc tissue is removed first, then the dura and spinal nerves may be finally exposed [7-9]. Differences between these two techniques are illustrated in Figure 2.

Outside-in technique

For patients who received the outside-in technique, the punctured site was taken 10-11 cm away from the medial spinous process, and a puncture needle was inserted targeting the ventral part of the SAP in the targeted intervertebral disc space. If the needle position was satisfactory, as confirmed with C-arm fluoroscopy, the punctured site was enlarged to 1.5 cm; then, the incision was gradually expanded with dilating tubes. Finally, the working cannula and spinal endoscope were inserted through the intervertebral foramen under visualization or not. The ventral and apical part of the SAP was removed (partial facetectomy was done depending on the space of foramen) with a bone drill or trephine to enlarge the intervertebral foramen, further exposing the traversing nerve root, dura sac, ligamentum flavum, annulus fibrosus, and herniated nucleus pulposus. The ruptured site of the annulus fibrosus was determined, and the protruded nucleus pulposus was removed. If the annulus fibrosus was not ruptured, a radiofrequency probe was used to burn a small incision at the most bulging part of the annulus fibrosus. The nucleus pulposus was sometimes squeezed out and removed appropriately. After the nerves were decompressed satisfactorily, the ruptured annulus fibrosus was coagulated with a radiofrequency probe. After sufficient hemostasis, the wound was closed with one full-thickness suture.

Inside-out technique

For patients receiving the inside-out technique, an incision was made 12-13 cm (generally larger than in the outside-in technique) away from the spinous process midline, and a needle was inserted into the targeted disc space approximately 20 sagittal degrees toward caudally. The position of the punctured needle was confirmed under fluorography, and methylene blue was sometimes injected into the disc to stain the degenerated nucleus. After serially expanding the incision through a guiding pin, a spinal endoscopy was inserted, and the nucleus was removed under observation. At this point, it was possible for the dura sac and traversing spinal nerve to be seen; however, in most cases, it was much harder to expose the nerve compared to the outside-in technique. Thus, it is difficult or even impossible to complete the foraminoplasty using the inside-out technique.

Statistical analysis

The homogeneity of the variances of data was tested by the Levene method with the SPSS 22.0 software (IBM Inc., Armonk, US) and then used to compare pre-operative and post-operative indicators using two-way repeated measures ANOVA. Percentage data were compared using the chi-squared test (X^2^). A p-value ≤0.05 signifies a significant difference.

## Results

**Figure 3:**
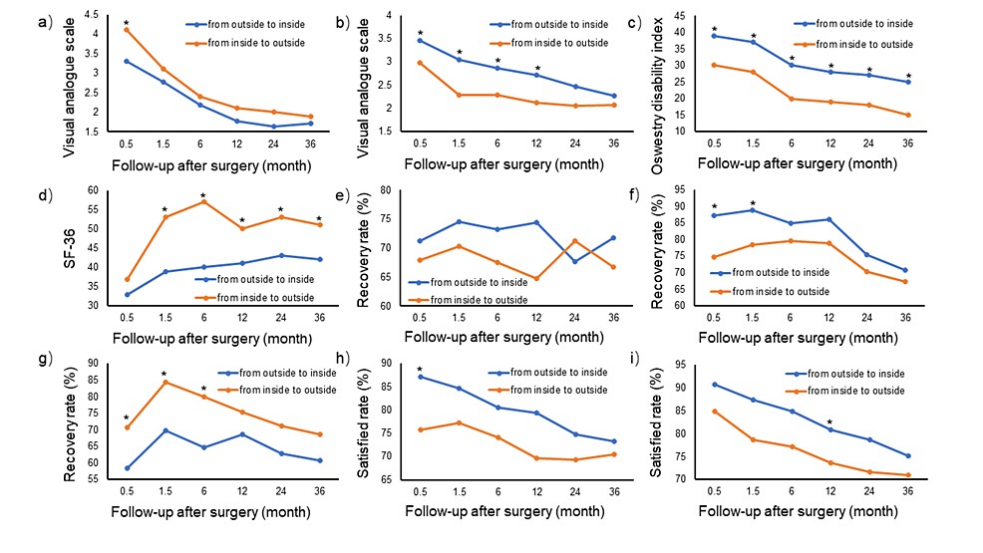
Changes in VAS score, ODI, SF-36 (physical component), pain recovery rate, and satisfaction rate with change and treatment before and after endoscopic discectomy surgery for both techniques at different time points (a) VAS for leg; (b) VAS for back; (c) ODI; (d) SF-36 (physical); (e) recovery rate from symptoms; (f) recovery rate from leg pain; (g) recovery rate from back pain; (h) satisfaction rate of change; (i) satisfaction rate of treatment. VAS - Visual Analogue Scale; ODI - Oswestry Disability Index; SF-36 - physical component of Short Form 36 Health Survey Questionnaire * indicates a significant difference (p≤0.05)

Five thousand cases were enrolled from three hospitals, comprising 3047 male patients and 1953 female patients, with an average age of 35.2±15.7 years. Characteristics of the patients are shown in Table 1, with all relevant data shown as means and standard deviations. There were no significant differences in the basic data between the two groups of patients. Operative time, blood loss, complications, and other related information for the two endoscopic lumbar discectomy techniques are shown in Table 2. There were significant changes in VAS, ODI, and SF-36 for both techniques just after surgery compared with pre-operative values (see Table 3 and Figure 3). Furthermore, there were significant differences in VAS for the leg, VAS for the back, ODI, and SF-36 at 0.5 months after surgery between the two techniques (see Table 3 and Figure 3).

**Table 1:** General information of participants Outside-in: one of the percutaneous transforaminal endoscopic lumbar discectomy (PTELD) techniques, processed from the extraforaminal space to the disc. Inside-out: one of the PTELD techniques, processed from the disc to the foraminal space. * Duration of radiating leg pain from onset until surgery. ^†^ VAS - Visual Analogue Scale; scores show the intensity of leg and back pain, ranging from 0 to 10, with higher scores indicating worse pain. ^‡^ ODI - Oswestry Disability Index; it was used to measure functional disability, ranging from 0 to 100, with higher scores indicating more functional disability. ^§^ SF-36 (physical) - physical component of Short Form 36 Health Survey Questionnaire, with higher scores indicating better quality of life. VAS, ODI, and SF-36 (physical) were all based on patient-reported questionaries. LDH - lumbar disc herniation

Characteristics	Outside-in	Inside-out	p-value
Cases	2039	1890	
Age (years; mean, SD)	41±24	38±30	0.25
Gender	Male (57%); female (43%)	Male (61%); female (39%)	0.37
Body mass index (mean, SD)	27±6	26±7	0.42
Duration of leg pain (months; mean, SD)*	6±3	5±4	0.06
Radiating to			
Left	39%	43%	0.71
Right	42%	39%	0.83
Both sides	19%	18%	0.64
Sensory deficit	45%	51%	0.49
Muscle weakness	31%	27%	0.36
Weakened tendon reflex in knees	13%	12%	0.65
Weakened tendon reflex in ankles	34%	41%	0.52
VAS for^ †^			
Leg (mean, SD)	6.4±3.2	6.1±4.5	0.75
Back (mean, SD)	5.7±2.6	5.9±3.8	0.88
ODI^‡^ (mean, SD)	49.4±21.7	50.5±19.3	0.79
SF-36 (physical score)^§^ (mean, SD)	28.3±11.8	30.7±12.4	0.42
Level of LDH			
L1/2	7.20%	6.80%	0.56
L2/3	6.50%	6.70%	0.43
L3/4	6.00%	7.10%	0.64
L4/5	41.10%	42.60%	0.55
L5/S1	39.20%	36.80%	0.41

**Table 2:** Information on surgery and intra-operative and post-operative complications of two endoscopic lumbar discectomy techniques * Blood loss was calculated by subtracting flushing normal saline fluid from the total collected fluid. P-value ≤0.05 indicates a significant difference. ^† ^If there is more than one area occupied by the herniated disc, the site where the largest protrusion is defined as the herniated site. ^‡^ Outside-in group: 92 (4.5%) patients had a pre-operative endoscope discectomy within one year, 37 (1.8%) patients received the same surgery in the following two years, and 18 (0.9%) patients had open fusion surgery during the follow-up. Inside-out group: 81 (4.3%) patients had a pre-operative endoscope discectomy within one year, 24 (1.3%) patients received the same surgery in the following two years, and 27(1.4%) patients had open fusion surgery during the follow-up. ^※ ^Signifies the patient started to walk 48 hours after surgery.

Surgery/complication	Outside-in	Inside-out	p-value
Time (min)	64±21	41±19	0.03
Blood loss (mL)*	73±38	39±14	0.01
Herniated site^†^			
Median	664 (32.6%)	519 (27.5%)	0.51
Paramedian	981 (48.1%)	939 (49.7%)	0.76
Intraforaminal	275 (13.5%)	214 (11.3%)	0.09
Extraformainal	119 (5.8%)	218 (11.5%)	0.02
Intraoperative complications			
Wrong level	58 (2.8%)	40 (2.1%)	0.04
Nerve root injury	90 (4.4%)	61 (3.2%)	0.03
Dura tear	110 (5.4%)	76 (4.0%)	0.04
Intracranial hypertension	44 (2.2%)	29 (1.5%)	0.07
Vessel injury	27 (1.3%)	29 (1.5%)	0.49
Intestine injury	11 (0.54%)	23 (1.2%)	0.01
Post-operative complications			
Worse neurology deficit	161 (7.9%)	62 (3.3%)	0.01
Surgery site infection	12 (0.6%)	11 (0.59%)	0.45
Hematoma formation	10 (0.49%)	18 (0.95%)	0.03
Cerebrospinal fluid leak	108 (5.3%)	65 (3.5%)	0.05
Bowel and urination disorder	14 (0.7%)	16 (0.85%)	0.66
Reoperation^‡^	147 (7.2%)	132 (7.0%)	0.37
Timing of mobilization			
Day of surgery	304 (14.9%)	347 (18.4%)	0.17
Day one after surgery	1473 (72.2%)	1465 (77.5%)	0.32
Day two after surgery^※^	262 (12.9%)	78 (4.1%)	0.01

**Table 3:** Comparison of median VAS scores for leg and back pain, ODI, SF-36 (physical) score, recovery rate, and satisfaction rate between outside-in and inside-out techniques at different time points Outside-in: one of the percutaneous transforaminal endoscopic lumbar discectomy (PTELD) techniques, processed from the extraforaminal space to the disc. Inside-out: one of the PTELD techniques, processed from the disc to the foraminal space. VAS - Visual Analogue Scale; scores show the intensity of leg and back pain, ranging from 0 to 10, with higher scores indicating worse pain. ODI - Oswestry Disability Index; it was used to measure functional disability, ranging from 0 to 100, with higher scores indicating more functional disability. SF-36 (physical) - physical component of Short Form 36 Health Survey Questionnaire, with higher scores indicating better quality of life. VAS, ODI, and SF-36 (physical) were all based on patient-reported questionaries.

Outcome	Preoperative		p	Two weeks		p	Six weeks		p	Six months		p	12 months		p	24 months		p	36 months		p
	after outside-in	after inside-out		after outside-in	after inside-out		after outside-in	after inside-out		after outside-in	after inside-out		after outside-in	after inside-out		outside-in	inside-out		after outside-in	after inside-out	
VAS scores: leg	6.4±3.2	6.1±4.5	0.75	3.31±1.07	4.1±2.28	0.04	2.78±1.82	3.1±1.79	0.06	2.18±1.72	2.4±1.83	0.32	1.78±1.45	2.1±1.34	0.12	1.63±1.17	2.0±1.72	0.08	1.71±1.35	1.9±1.80	0.37
VAS scores: back	5.7±2.6	5.9±3.8	0.88	3.47±1.86	2.97±1.74	0.03	3.05±2.17	2.29±1.84	0.04	2.87±1.84	2.27±1.34	0.03	2.71±1.89	2.11±1.24	0.05	2.46±1.75	2.05±1.35	0.06	2.27±1.81	2.07±1.11	0.14
ODI	49.4±21.7	50.5±19.3	0.79	39±18	30±14	0.03	37±16	28±19	0.02	30±17	20±13	0.03	28±14	19±16	0.04	27±14	18±15	0.04	25±18	15±19	0.02
SF-36 (physical)	28.3±11.8	30.7±12.4	0.42	33±8	37±10	0.47	39±11	53±15	0.05	40±18	57±10	0.04	41±9	50±14	0.03	43±12	53±17	0.05	42±14	51±19	0.04
Recovery from symptoms (%)				71.2	67.9	0.39	74.6	70.3	0.69	73.2	67.6	0.47	74.4	64.8	0.5	67.7	71.2	0.44	71.8	66.7	0.63
Recovery from leg pain (%)				87.2	74.8	0.03	88.7	78.4	0.05	84.9	79.6	0.47	86.1	78.8	0.7	75.3	70.4	0.19	70.7	67.4	0.34
Recovery from back pain (%)				58.3	70.8	0.02	69.7	84.4	0.03	64.7	79.9	0.05	68.6	75.3	0.09	62.7	71.2	0.27	60.8	68.7	0.39
Satisfied with change (%)				87.1	75.8	0.05	84.6	77.2	0.08	80.5	74.1	0.34	79.4	69.6	0.07	74.8	69.3	0.11	73.2	70.4	0.34
Satisfied with treatment（%）				90.7	84.8	0.29	87.4	78.6	0.41	84.9	77.2	0.56	80.8	73.7	0.05	78.6	71.7	0.08	75.2	70.9	0.12

In detail, the outside-in group had significantly better relief from leg pain in terms of VAS than the inside-out group at 0.5 months after surgery, as well as a better recovery rate from leg pain and satisfaction rate (Table 3 and Figure 3). However, back pain VAS was better in the inside-out group than in the outside-in group, and from the viewpoint of functional evaluation, ODI and SF-36 were the same at 0.5 months after surgery. Then, there were no significant differences in VAS for leg pain during follow-up, whereas significant differences existed between the two technique groups for VAS for back pain from 0.5 months to 12 months post-operatively, as well as during follow-up for ODI and SF-36.

VAS, ODI, and SF-36 steadily improved within six months in both PTELD types; after that, the above parameters did not improve significantly. VAS for leg and back, ODI, and SF-36 all improved significantly after surgery; however, the above-evaluated scores were all significantly different between the outside-in and inside-out groups. In detail, the outside-in patients suffered more back pain and worse ODI and SF-36 but had more relief from leg pain 0.5 months after surgery in terms of VAS. As for recovery rate from symptoms, the two techniques were not significantly different, even though there were significant differences in recovery rate for leg pain and back pain at the first 1.5 months after surgery. As for satisfaction with the treatment, the outside-in technique had better results than the inside-out technique at 0.5 months and 12 months, whereas there was no significant difference at other time points.

## Discussion

It is well known that facet integrity plays an important role in stabilizing the spine during movement, and more than half of facet joint injuries will lead to significant dynamic instability [15]. Facetectomy is a common procedure that is performed with the outside-in PTELD technique. However, few studies have been published on this vital issue. Therefore, this study aimed to evaluate the effects of the outside-in and inside-out techniques.

In this study, the surgical position did not influence clinical effects, even though some surgeons prefer one position over the other. This study showed that both techniques are effective in relieving radiculopathy symptoms of the lower extremities. However, the outside-in technique relieved leg pain better than the inside-out technique at two weeks post-operatively, after which the significant difference subsided. This was most likely because the foramen space and ventral side of the nerves were well exposed, allowing for better decompression. As an opposing effect, facetectomy and destruction of the annulus fibrosus may jeopardize the segmental stability of the surgical level, which decreases the effect of decompression. In contrast, as the inside-out technique does not enlarge the foramen space and expose the nerves before removing the disc tissue, sufficient decompression may not be achieved. Therefore, we cautiously recommend that the outside-in technique is indicated for LDH with foraminal stenosis, whereas the inside-out technique is indicated for pure LDH.

Patients in the outside-in group suffered more back pain from two weeks post-operatively to one-year follow-up. This was most likely because facetectomy destabilized the surgery segment, resulting in more back pain until enough scar tissue formed one year later. This was also true for the quality of life between the two groups, possibly for the same reason. ODI and SF-36 both indicated better results from the inside-out technique compared to the outside-in.

Although both are minimally invasive surgeries, the outside-in technique took more time and led to more bleeding than the inside-out technique. Furthermore, both are prone to some complications [16]. Our results showed an incidence of intra-operative complications of 16.4% for the outside-in technique, whereas that of the inside-out technique was less (13.5%), with significant differences observed with wrong level, nerve root injury, dura tear (outside-in suffered higher incidence), and intestine injury (inside-out suffered significantly higher incidence). This may be due to the complicated procedure used to expose the dura and nerves in the outside-in technique, whereas the inside-out technique generally requires a bigger coronal abduct angle, thus resulting in a higher incidence of puncturing into the abdomen.

For post-operative complications, patients in the outside-in group suffered a higher incidence of worse neurology deficit and cerebrospinal fluid leak, whereas patients in the inside-out group suffered more hematoma formation. This is also because the outside-in technique involves more procedures to expose the dura sac and nerve roots, increasing the risk of nerve injury and cerebrospinal fluid leak. For the inside-out technique, a puncture needle was inserted through paraspinal muscles with a bigger abductive angle, possibly leading to an increased risk of retroperitoneal and paraspinal hematoma formation. As for the reoperation rate and incidence of surgical site infection, there were no significant differences between the two techniques. The main reasons for reoperation were relapse, failure of disc removal, and hematoma formation.

PTELD may not impede the progress of spine degeneration, and it may even make it worse in some cases [17]. The outside-in technique generally requires the removal of part of the SAP, which may destabilize the lumbar spine during movement, resulting in accelerated degeneration [18]. Even though the incidence of instability being higher in the outside-in group was expected, the evidence of instability was not well supported by data or images in this study. One of the reasons for this is that the dynamic balance of the spine is hard to evaluate concisely with current medical images. Furthermore, both endoscopy discectomy techniques will break the disc tissue and worsen its degeneration, which may partly explain the residual back pain two years after surgery.

This study did have some limitations. First, we did not set precise controls for surgeons. Second, we did not analyze whether the type of disc (medio/lateral/far lateral) also had an influence on the outcome, which may influence the results. Therefore, studies with a higher level of evidence are required to evaluate the long-term effectiveness of these techniques in the future.

## Conclusions

This is one of the few studies to evaluate different transforaminal discectomy techniques. Our results showed that both techniques were effective; the outside-in technique achieved better decompression but increased the risk of low back pain because of facetectomy, thus significantly influencing quality of life. Therefore, we should pay attention to preserving the facet and other surrounding normal tissues to avoid instability and tissue degeneration.
